# *In Silico* Assay Development for Screening of Tetracyclic Triterpenoids as Anticancer Agents against Human Breast Cancer Cell Line MCF7

**DOI:** 10.1371/journal.pone.0111049

**Published:** 2014-11-03

**Authors:** Om Prakash, Ateeque Ahmad, Vinay Kumar Tripathi, Sudeep Tandon, Aditya Bhusan Pant, Feroz Khan

**Affiliations:** 1 Metabolic and Structural Biology Department, CSIR-Central Institute of Medicinal and Aromatic Plants, Lucknow, Uttar Pradesh, India; 2 Process Chemistry & Chemical Engineering Department, CSIR-Central Institute of Medicinal and Aromatic Plants, Lucknow, Uttar Pradesh, India; 3 In-vitro Toxicology Division, CSIR-Indian Institute of Toxicology Research, Lucknow, Uttar Pradesh, India; University of Ulm, Germany

## Abstract

Experimental activity of a compound on cancer cell line/target is mostly analyzed in the form of percentage inhibition at different concentration gradient and time of incubation. In this study a statistical model has been developed referred as *in silico* assay using support vector regression model, which can act with change in concentration gradient and time of incubation. This model is a function of concentration gradient, treatment hour and independent components; which calculate the percentage inhibition in combination of above three components. This model is designed to screen tetracyclic triterpenoids active against human breast cancer cell line MCF7. The model has been statistically validated, checked for applicability domain and predicted results were reconfirmed by MTT assay, for example Oenotheranstrol derivatives, OenA & B. Computational SAR, target and docking studies were performed to understand the cytotoxic mechanism of action of Oenotheranstrol compounds. The proposed *in silico* assay model will work for specific chemical family for which it will be optimized. This model can be used to analyze growth kinetics pattern on different human cancer cell lines for designed compounds.

## Introduction

*In vitro* experiment is essential for screening of small molecules active against specific target/cell line. Normally, different human cancer cell lines are used in *in vitro* experiment for the screening of anticancer compounds [1]. Similarly, in virtual screening, screening of compounds is performed by quantitative structure-activity relationship (QSAR) approach to predict the possible activity. In general, QSAR models do not predict the compound's percentage inhibition, in accordance to concentration gradient and time of incubation. These limitations of QSAR model mostly restrict the prediction of low activity compounds. To overcome these barriers, support vector regression (SVR) model (*in silico* assay) has been proposed.

In the present study, supervised learning based model is used for *in silico* assay. The supervision to the model has been provided in the form of percentage inhibition. *In silico* assay can be considered as evolved QSAR model, in which the experimental parameter variations have been compiled in combination of structural information. Multi-parameter based experimental dataset has a good level of non-linearity. Algorithms e.g. artificial neural network (ANN) and support vector machine (SVM) are efficient to cover the non-linearity of the input data by implementing the kernel functions. Kernel functions are used to map high dimensional feature vectors. Beside these algorithms, other methods like: Hidden Markov Models (HMM), Hierarchical Bayesian Networks (HBN), Bayesian Networks (BN) etc. are also known for modeling. In this study, SVM based non-linear modeling has been performed. SVM is originated from statistical learning theory of structural risk minimization principle. It provides an efficient facility of demarcation of boundary conditions in the form of support vectors. Support Vector Regression (SVR) is well known for patterns recognition relationship establishment in supervision of respective empirical target [2]. During screening studies, initially the cellular targets for query compounds are not known. It is also not known, which molecular fragment is responsible for activity. To perform such studies through computational means, database mining based SAR and target identification is one of the best ways. MetaDrug (Thomson Reuters, USA) provides data mining facility to search out possible targets, metabolite generation and concerned reactions, network analysis, gene ontology and possible toxicity report.

Triterpenoids are known for their cytotoxic and anti-inflammatory activity from plant sources [3], [4]. Terpenoids are divided into many groups on the basis of their structures. Oenotheranstrol derivatives belong to T-type triterpenoids, which is a group of tetracyclic triterpenoids [5]. The present work for development of *in silico* assay model using support vector regression method is presenting a new theme of understanding the computational assay. The assay model has been optimized and validated for tetracyclic triterpenoid series of compounds. A case study has been performed with OenA and OenB compounds. Testing of OenA and OenB based 48 samples has been performed against the proposed *in silico* assay model. The OenA & OenB has been experimentally validated through *in vitro* MTT assay. This case study was performed with OenA & B, because the *in silico* assay model has been optimized for tetracyclic triterpenoids acting against human cancer cell line MCF7.

## Materials and Method

**Figure 1:**
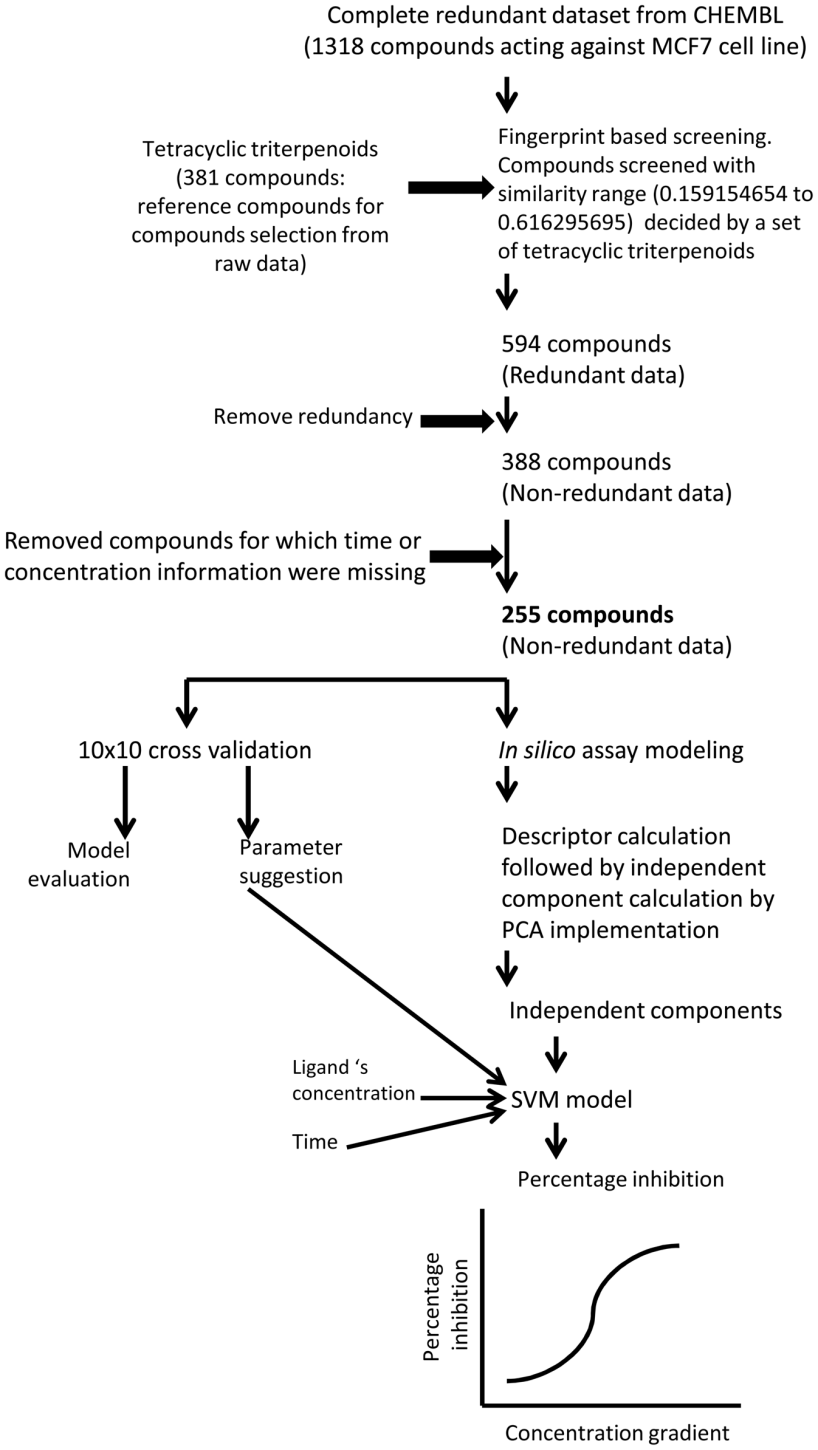
Overall workflow chart for in silico assay model building.

Overall workflow for *in silico* assay development process has been summarized in pictorial representation (Figure 1). The *in silico* assay modeling hypothesis was evaluated by statistical validation, applicability domain check and a case study. The case study was performed with two query compounds OenA & B. Case study has been experimentally validated through *in vitro* assay. Further SAR analysis and exploration of mechanism of action of OenA & B were also described.

### Data preparation

**Figure 2:**
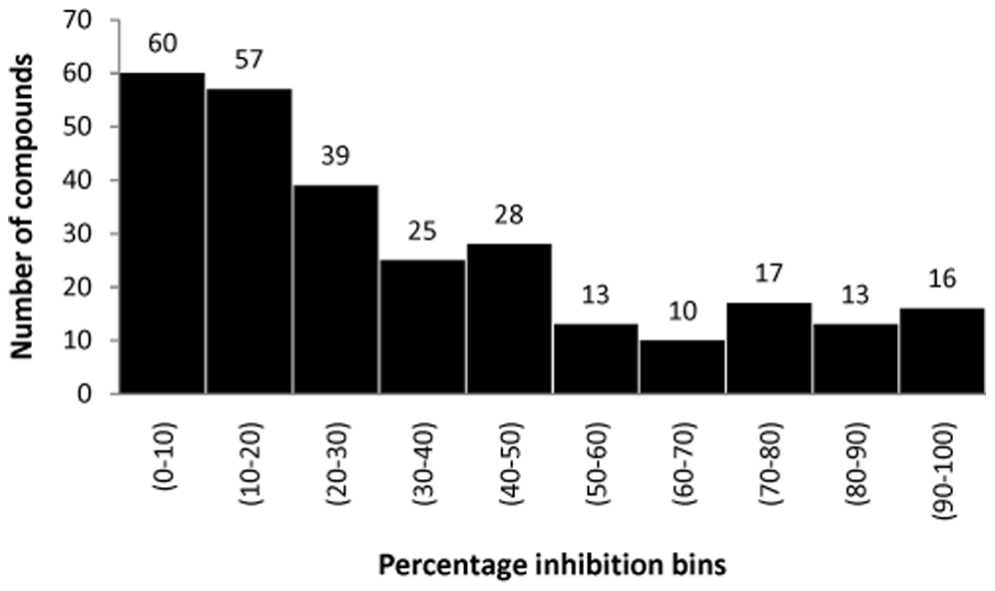
Histogram of training dataset, showing distribution of percentage inhibition from zero to 100%.

The complete set of redundant raw data (1318 compounds) for percentage inhibition of MCF7 human cancer cell line were collected form CHEMBL database [2] (data S1). Since, the aim of the present study was to develop *in silico* assay model for tetracyclic triterpenoids for screening of anticancer compounds against human breast cancer cell line MCF7. Therefore a set of available 381 tetracyclic triterpenoids were collected from ChEBI database (data S2). The set of tetracyclic triterpenoids was used to build a profile based on their molecular fingerprints. This profile was used to select the compounds from set of 1318 compounds. After removal of redundancy, only 255 unique compounds were selected for model development (data S3). The selected set of 255 compounds includes experimental records in the terms of percentage inhibition, treatment concentration (µM) and incubation duration (Hours). The compounds were grouped on the basis of their percentage inhibition against MCF7 cancer cell line. Histogram for training dataset of 255 compounds with percentage inhibition has been shown into Figure 2.

### Independent descriptor components and *in silico* assay model development

Two dimensional molecular descriptors for 255 molecules were calculated using CDK library [http://rguha.net/code/java/cdkdesc.html]. The calculated 270 molecular descriptors belong to five major categories: electronic, topological, geometrical, constitutional and hybrid types. These chemical descriptors were processed through principal component analysis to transform the molecular descriptors into independent components. The useful independent components were selected by backward elimination method [2]. Since there was high dimensionality in the dataset, therefore normalization was performed in the range of −1.0 to 1.0. The selected independent components along with treatment concentration (µM) and duration (Hours) values were used in LibSVM [6] for *in silico* assay model building. The *in silico* assay is a function of concentration gradient, treatment gradient and independent components, which calculate the percentage inhibition in combination of three components. 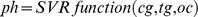



Where, ph, cg, tg and oc are ‘percentage inhibition’, ‘concentration gradient’, ‘treatment gradient’ and ‘independent components’ respectively. Conclusively, percentage inhibition is the only output of the model. To find out best LibSVM parameter combinations, grid based parameter selection algorithm has been applied with ‘gamma’ and ‘cost’ values. In this process, efficiency of each model against each parameter combination was analyzed to achieve the best parameter combination. Protocol for *in silico* assay model development (LibSVM based) has been shown in Figure 1.

### Kernel function selection for model development

Kernel functions are the most essential part of machine learning based methods. SVM supports linear, polynomial, sigmoid and radial basis kernel functions for non-linear modeling. To select the most efficient kernel function for model development, the existing non-linear kernel functions were applied with training dataset along with their parameter combinations. Two parameters (gamma  = 0 and cost  = 1) were selected for model evaluation. A comparison has been made to select the most efficient kernel function with support vector regression model for given dataset. Details are tabulated in data S4.

### Cross validation of model and parameter selection

The 10×10 cross-validation was used to characterize the potential risk of biasness in data dependent parameter selection. For validation, overall training dataset of 255 compounds were divided into 10 sets. Nine sets were used for adapting the regression model and tenth set was used for testing of adapted model. The procedure was repeated for all 10 different splits. Each run of 10×10 fold cross validation include the construction of the principal components. The training process for each single model also includes the parameter selection process. Optimal values of Gamma and Cost were estimated by grid based parameter procedure. For optimal parameter selection, the Gamma and Cost values were considered between 10^−5^ to 1.0 and 0.01 to 10^5^ respectively. The model development and cross validation was performed with identified efficient radial basis kernel function. The optimized parameters were used for selection of best *in silico* model. In each set of cross validation, 100 models were developed. These 100 models were evaluated on the basis of following criteria:
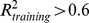


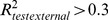



### Compounds used in experimental validation of model

The compounds, Oenotheranstrol-A (Lanosta-5-en-2β, 3β, 26, 27-tetraol-21-oic acid) and Oenotheranstrol-B (Lanosta-5-en-2β, 3β, 26, 30-tetrol-21-oic acid) from the *Oenothera biennis* roots were used for experimental validation of model [7] [Appendix S1].

### Applicability domain (AD) check using molecular descriptors and experimental parameters

Applicability domain was defined by the 255 inhibitors of MCF7 cell line used in bioassay model building. AD was designed in the form of range value of Lipinski's parameters, topological polar surface area, ligand concentration, treatment time and percentage inhibition. The input parameters for query compounds were screened through applicability domain decided by training set.

### *In vitro* experimental validation of *in silico* assay by MTT and LDH assay

#### MTT assay

Non-cytotoxic dose of OenA and B was identified in HaCaT and MCF7 human cell lines. Cytotoxicity assessment was done using standard endpoint i.e., tetrazolium bromide MTT (3-(4, 5-dimethylthiazol-2-yl)-2, 5-diphenyl tetrazolium bromide) assay fallowing the protocol of Kashyap *et al* 2010 [8]. In brief, cells (1×10^4^ cells/well) were seeded in 96-well tissue culture plates and incubated in the CO_2_ incubator for 24 h at 37°C. Then the medium was aspirated and cells were exposed to medium containing OenA and B (0.001 to 100 µg/ml) for 24–96 h at 37°C in 5% CO_2_-95% atmosphere under high humid conditions. Tetrazolium salt (10 µl/well; 5 mg/ml of stock in PBS) was added 4 h prior to completion of respective incubation periods. At the completion of incubation period, the reaction mixture was carefully taken out and 200 µl of culture grade DMSO was added to each well. The content was mixed well by pipetting up and down several times until dissolved completely. Plates were then incubated for 10 minutes at room temperature and color was read at 550 nm using Multiwell Microplate Reader (Synergy HT, Bio-Tek, USA). The unexposed sets were also run parallel under identical conditions that served as a basal control.

#### LDH release assay

Lactate dehydrogenase (LDH) release assay is a method to measure the membrane integrity as a function of the amount of cytoplasmic LDH released into the medium. The assay was carried out using readymade commercially available LDH assay kit for *in vitro* cytotoxicity evaluation (TOX-7, Sigma St. Louis, MO., USA). The assay was based on the reduction of NAD by the action of LDH. The resulting reduced NAD (NADH^+^) was utilized in the stoichiometric conversion of a tetrazolium dye. The resulting colored compound was measured using multiwell plate reader at wavelengths 490 and 690 nm. In brief, the cells were exposed with (0.001 to 100 µg/ml) for different time periods after the completion of respective time periods the cells were processed for LDH release assay similar to MTT assay. Culture plates were removed from CO_2_ incubator as per the experimental schedule and centrifuged at 250×g for 4 min. Then supernatant of each well was transferred to a fresh flat bottom 96 well culture plate and processed further for enzymatic analysis as per the manufacturer's instructions.

The results of MTT assay and *in silico* bioassay were compared to validate the model results. Validation was performed with analysis of percentage inhibition against compound's concentration gradient of ‘0.001 to 100 µg/ml’ and readings were compared at 24, 48, 72 and 96 hours.

### *In silico* studies for SAR, possible target identification and mechanism of action

Training dataset analysis, MetaDrug data mining (Thomson Reuters, USA) and background information about tetracyclic triterpenoids were used to extract information about structure activity relationship of query compounds, their possible target in MCF7 cell line, possible toxicity and mechanism of action. The identified possible target (Cytochrome P450 19A1; PDB: 3EQM) was used for docking studies with OenA & B and control compound androstenedione. Co-crystallized tetracyclic triterpenoid Androstenedione with CYP19A1 was used for docking program validation. Molecular docking was performed by ‘AutoDock Vina’ software [9].

## Results

### RBF kernel function in *in silico* assay

**Figure 3:**
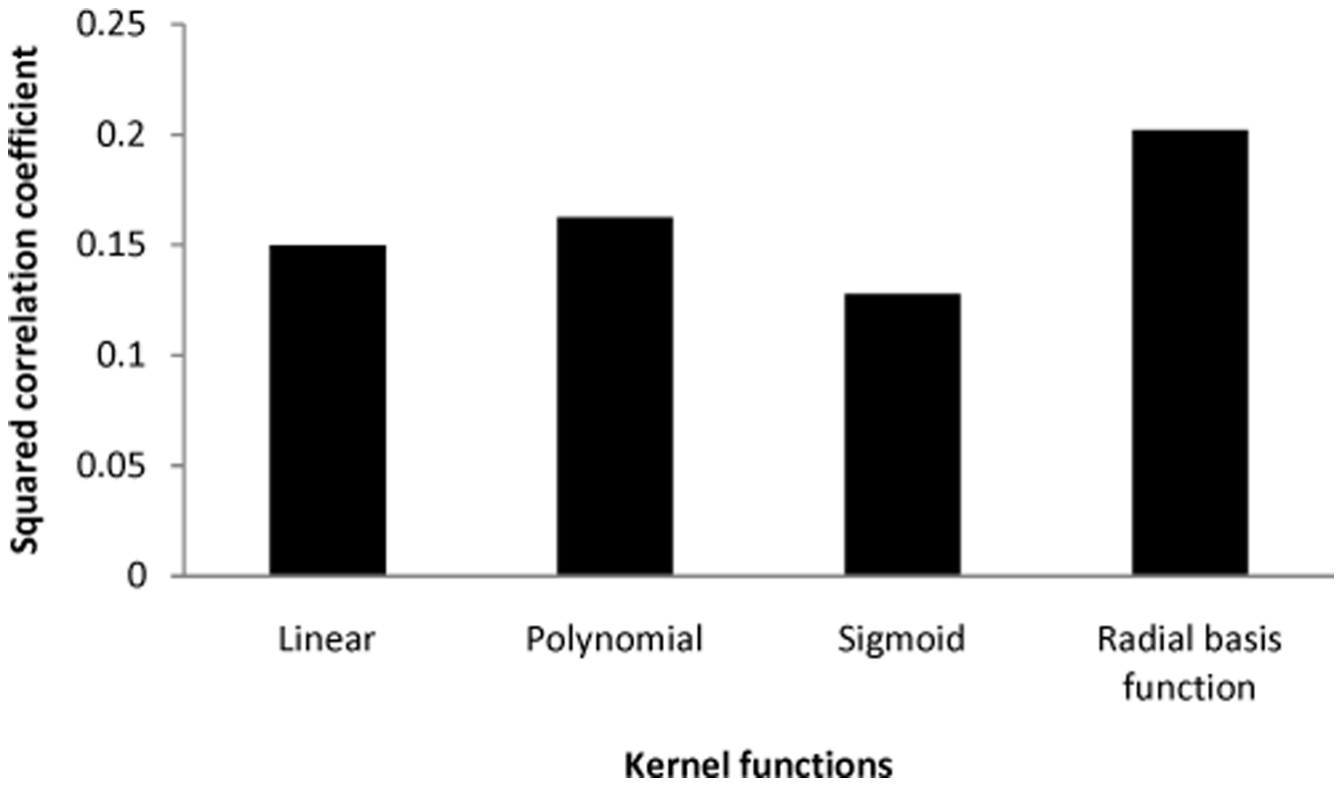
Comparison of efficiency of kernel functions for regression modeling with given dataset.

The three kernel functions were compared for efficiency for SVM model development. RBF was observed to perform more efficient in comparison of other kernel functions. Figure 3 shows comparison of SVM model's efficiency with four different kernel functions. The graph motivates for implementation of RBF function for model development.

### *In silico* assay model

Epsilon-SVR algorithm with radial basis kernel function was used for model building [2]. Model has been optimized for two parameters ‘gamma’ and ‘cost’ [2]. ‘Gamma’ (ranging between 10^−5^ and 1) and ‘Cost’ (ranging between 0.01 and 10^5^) factors were used in model optimization (data S5). Other parameters were considered at the software's default as: epsilon: 0.1; termination criterion tolerance: 0.001; rho: 0.697996; shrinking heuristics was used and initial data was normalized between −1.0 to 1.0. Total 100 parameter combinations (10 sets of Gamma and 10 sets of Cost values) were used during the model optimization. The developed 100 models were analyzed on the basis of optimized parameter combinations obtained from cross validation experiments (Table 1). It was observed that in 77.78% cases where cross validation sets are following the model evaluation criteria (R^2^_training_>0.6 and R^2^_test external_>0.3), contains the ‘Cost’ value of 11111.1. While the above model evaluation criteria was followed by only 33.33% cases with ‘Gamma  = 0.55556’. Consistency in regression coefficient value for test sets shows the unbiased nature of dataset for model building. Therefore, the *in silico* assay models were evaluated on two basic criteria: R^2^_training_>0.6 and Cost  = 11111.1 along with lowest ‘Gamma’ value for finest function approximation.

**Table 1:** 10×10 cross validation for evaluation of unbiased nature of dataset used for *in silico* assay development.

Set no.	Number of compounds in training set	Number of independent components*	Number of compounds in test set	Gamma	Cost	R^2^_training set_	R^2^_external test set_	RMSE	Q^2^
1	230	08	25	0.33334	11111.1	0.768892	0.338021	13.6271	0.768221
2	229	06	26	0.55556	44444.5	0.837094	0.305611	11.9228	0.836886
3	229	06	26	0.55556	11111.1	0.723818	0.315206	15.3982	0.719873
4	229	07	26	0.11112	66666.7	0.680726	0.282882	16.2849	0.672414
5	229	07	26	0.77778	33333.3	0.769145	0.308949	13.819	0.766843
6	230	05	25	0.77778	11111.1	0.623196	0.409164	17.5192	0.620397
7	230	08	25	1	11111.1	0.879962	0.333284	9.68034	0.879639
8	230	07	25	0.66667	11111.1	0.843096	0.34376	11.4614	0.841424
9	230	06	25	0.77778	11111.1	0.727246	0.476459	14.9093	0.722103
10	229	06	26	0.77778	11111.1	0.732883	0.354415	15.1167	0.729547

*Including, concentration and time factor.

**Figure 4:**
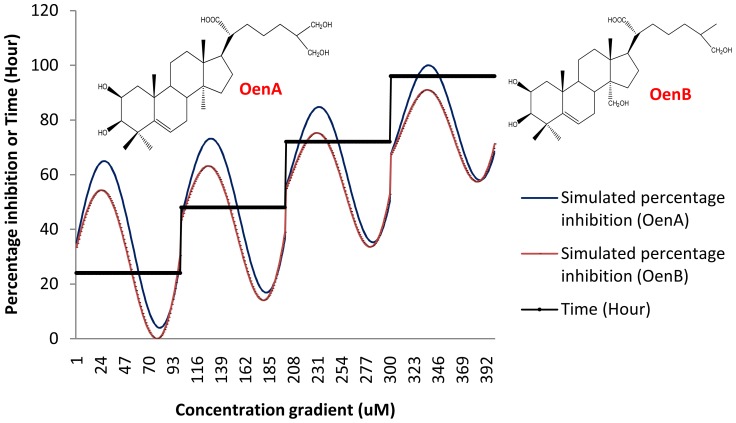
Simulated in silico assay model plot for two compounds OenA and B within a range of concentration gradient (0.01 to 500 µM) at four different time intervals of 24, 48, 72 and 96 hours.

By implementing above criteria, most efficient *in silico* model was found with R^2^ = 0.687115, Gamma  = 0.11112, Cost = 11111.1 and 200 support vectors. Details about 200 support vectors have been shown in ‘data S6’. The assay model function was used to calculate the percentage inhibition value for compounds (OenA & B) with concentration and time gradient, The simulated *in silico* assay model has been shown in Figure 4 for a range of concentration gradient (0.01 to 500 µM) of OenA & B at four different time intervals of 24, 48, 72 and 96 hours. Resultant graph of *in silico* assay model followed the cell culture growth curve (at y-axis: percentage inhibition at 24, 48, 72 and 96 hours) with retardation effect due to concentration of tested compound. These curves are the resultant expressed from the model (support vector regression model equation developed with independent components, concentration and time). The graph represents the success of model building process.

The developed regression models can be used for prediction of activity for query compound. It is based on support vector regression model with 200 support vectors for decision making. Since we used the experimental activity of the training set compounds in the form of percentage inhibition against MCF7 cancer cell line, therefore model is predicting the same based on some variables of the model. Percentage inhibition is the only output of the model.

### Experimental validation of *in silico* assay model: A case study using OenA & B compounds

**Figure 5:**
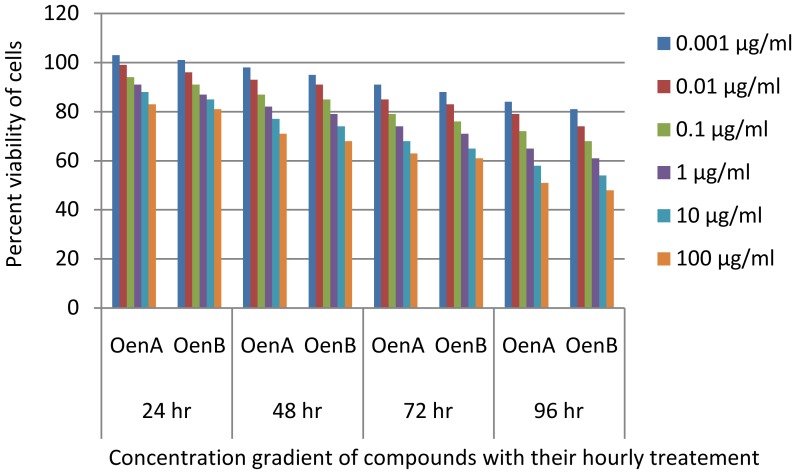
Graph between percentage viability & compound treatment at 24, 48, 72 & 96 hours based on MTT assay (with MCF7 cancer cell line).

**Figure 6:**
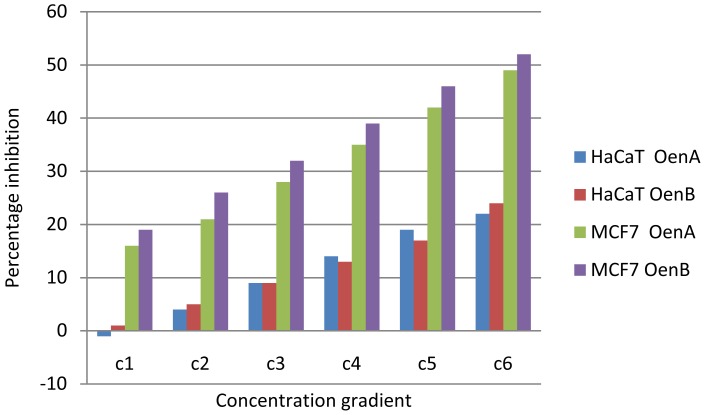
Comparative bioassay (MTT) results for normal cell line (HaCaT) & MCF7 cancer cell line at 96 hour. Results show the significant difference made by the treatment with OenA & B on normal and cancer cell lines. This difference indicates the existence of anticancer property of the Oenotheranstrol derivatives against human cancer cell line MCF7.

**Figure 7:**
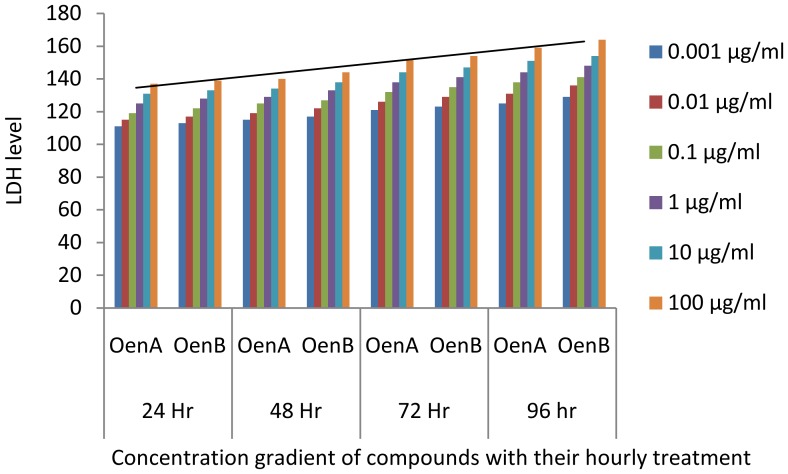
Graph between LDH level & compound treatment at 24, 48, 72 & 96 hours based on LDH release assay (with MCF7 cell line).

In MTT assay, all the cells responded significantly to all the chemicals tested in a dose dependent manner. Cells exposed to OenA and B (0.001–1 µg/ml) have shown no significant reduction in percent cell in 96 hrs. Whereas, higher concentrations of OenA and B used i.e., 10 and 100 µg/ml were found to cause gradual reduction in percent cell viability, which reaches to significant levels at and above 48 h exposure (Figure 5). Treatment of ligands with normal cell line HaCaT didn't showed significant level of inhibition (Figure 6). In LDH release assay, the highlights of the results for the release of lactate dehydrogenase following the exposure of compound A and B are presented in Figure 7. A significant increase in LDH release was observed in 10 and 100 µg/ml in comparison to control in all the cell lines.

Applicability domain (AD) was defined by the training set compounds containing: percentage inhibition (varying between 0 and 103); ligand concentration (0.05 to 230.1708); treatment time (Hr) (ranging between 3 to 168); TPSA (0 to 282.47 Å^2^); number of hydrogen bond acceptors (1 to 25); number of hydrogen bond donors (0 to 2); molecular weight (105 to 1145.979) and LogP (−4.898 to 12.314 -). All parameter combinations of query molecules (OenA & B) were found within applicability domain except small variation in case of initial ligand concentration and number of hydrogen bond donors. Percentage inhibition was found ranging from 6.55373 to 50.6219 by treatment of ligands with concentration gradient (0.001976 to 197.628 µM) for 24 to 96 hours. Following Lipinski's parameters and TPSA for OenA & B were, TPSA = 17.07; number of hydrogen bond acceptors  = 6; number of hydrogen bond donors  = 4; molecular weight  = 506.724 and LogP  = 4.554. Results showed that, all the query combinations were found within the applicability domain (Table 2, 3).

**Table 2:** Applicability domain defined by the training dataset of 255 inhibitors active against MCF7 cancer cell line.

	Percentage inhibition	Concentration (µM)	Time (Hr)	Number of H-bond acceptors	Number of H-bond donors	Topological Polar Surface Area (Å^2^)	Molecular weight	LogP
Minimum value	0	0.05	3	1	0	0	105.9929	−4.898
Maximum value	103	230.1708	168	25	2	282.47	1145.979	12.314

**Table 3:** Applicability domain check for query molecules OenA and B.

Compound	Range	Predicted Percentage Inhibition	Concentration (µM)	Time (Hr)	Number of H-bond acceptors	Number of H-bond donors	Topological Polar Surface Area (Å^2^)	Molecular weight	LogP
OenA	Min. value	6.55373	0.001976	24	6	4	17.07	506.724	4.554
	Max. value	50.6219	197.628	96					
OenB	Min. value	6.55373	0.001976	24	6	4	17.07	506.724	4.554
	Max. value	50.6219	197.628	96					

In case study, 48 queries of two compounds OenA & B were used for experimental validation. Six point concentration gradient (0.001, 0.01, 0.1, 1, 10 and 100 µg/ml) and four point time gradient (24, 48, 72 and 96 hours) along with independent components were used in model testing. The query set was not involved in training of assay model. Query set expressed the significant result with R^2^ of 0.728251 (Table 4).

**Table 4:** Details about *in silico* assay model vs. experimental validation.

Number of compounds in training set	Number of independent components*	Gamma	Cost	R^2^_training_	R^2^_experimental validation_	RMSE	Q^2^
255	8	0.11112	11111.1	0.687115	0.728251	16.0918	0.685441

*Including, concentration and time factor.

**Figure 8:**
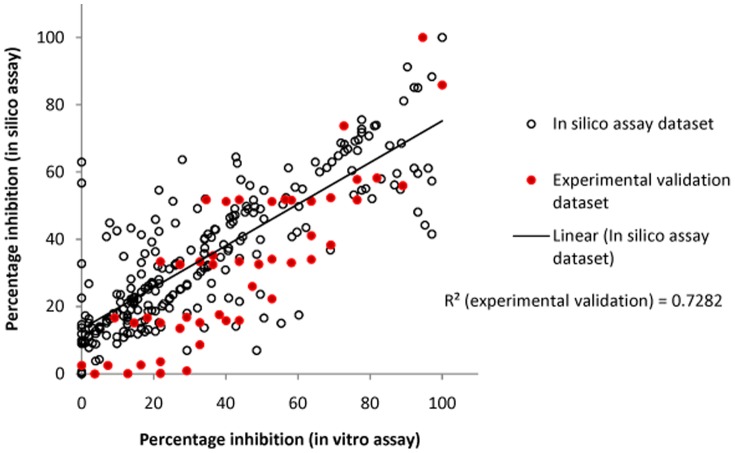
Combined (model vs. experimental) regression plot.

**Figure 9:**
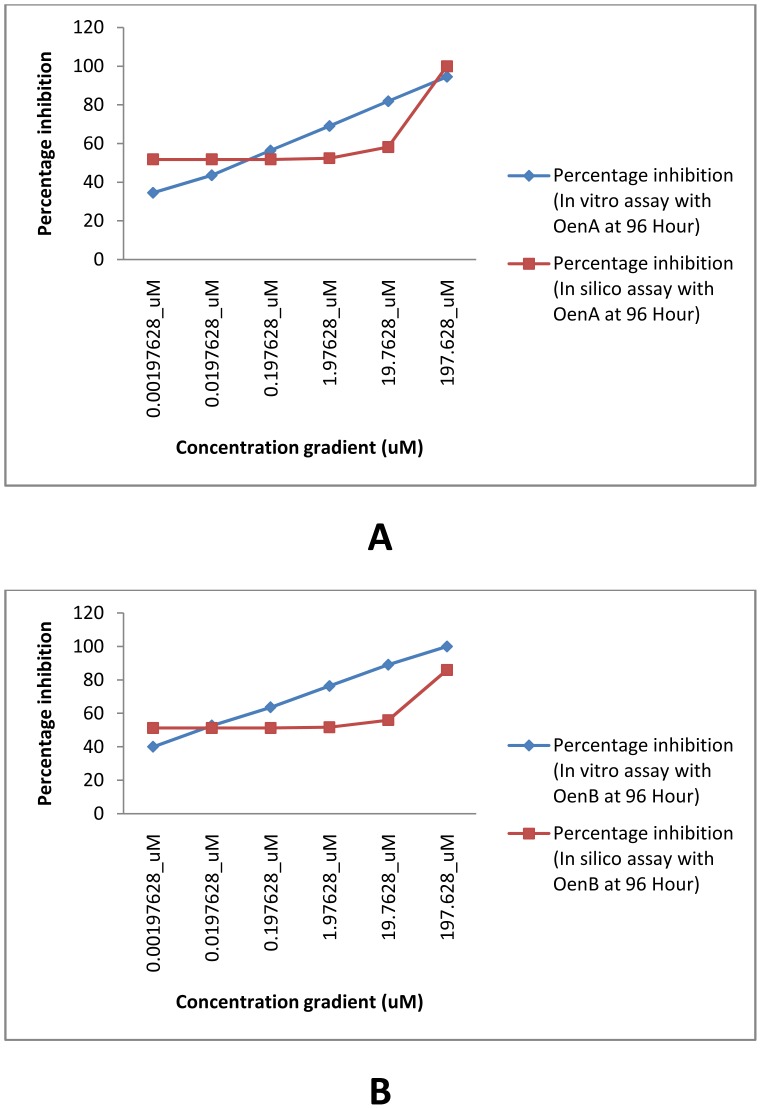
Graph A (for OenA) and B (for OenB) showing concentration gradient wise variation in percentage inhibition.

**Figure 10:**
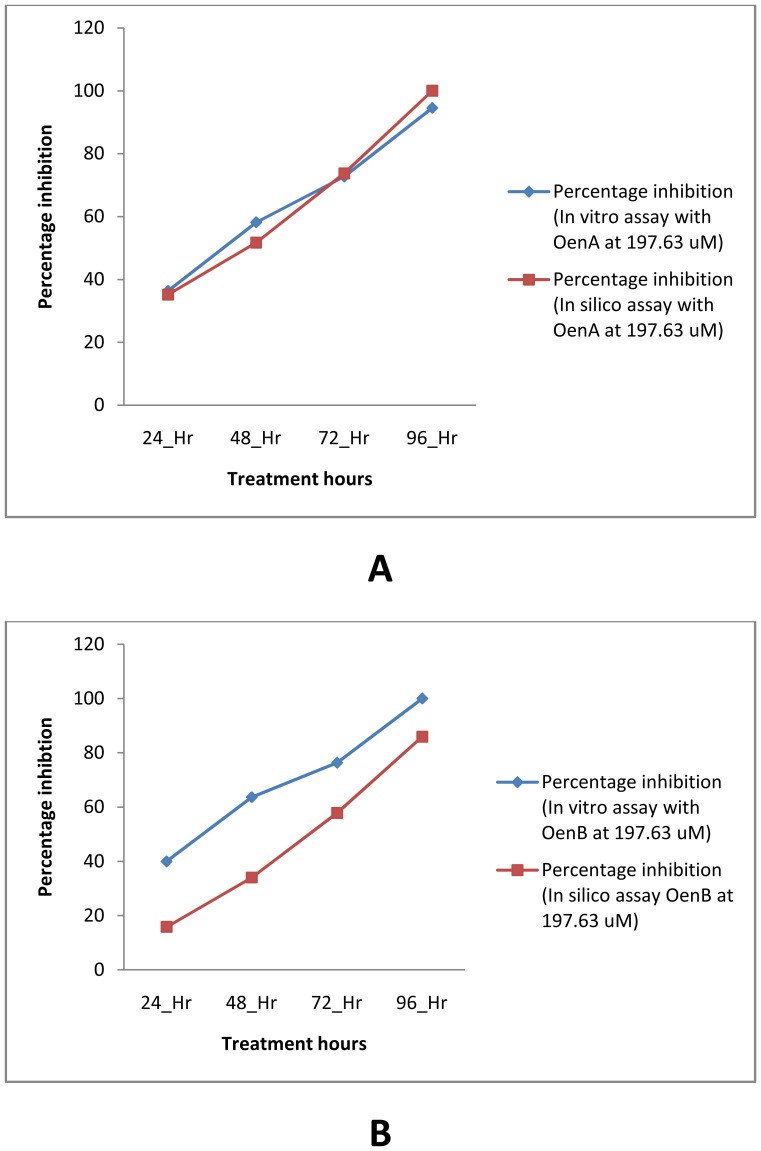
Graph A (for OenA) and B (for OenB) showing time gradient wise variation in percentage inhibition.

The overall correlation coefficient for *in silico* percentage inhibition vs. *in vitro* percentage inhibition for OenA & B compounds was 0.7282 (Figure 8). Compliance was observed between *in silico* assay results and *in vitro* assay results. In Figure 9 concentration gradient wise variation in percentage inhibition has been analyzed in relation with time of treatment at 96 hours. Compliance was observed between *in silico* assay and *in vitro* assay based percentage inhibition. Similarly, in Figure 10 ‘hour wise variation’ in percentage inhibition was analyzed at highest experimental concentration of 197.63 µM. Compliance was also found between *in silico* assay and *in vitro* assay.

### *In silico* studies for SAR (structure activity relationship)

**Figure 11:**
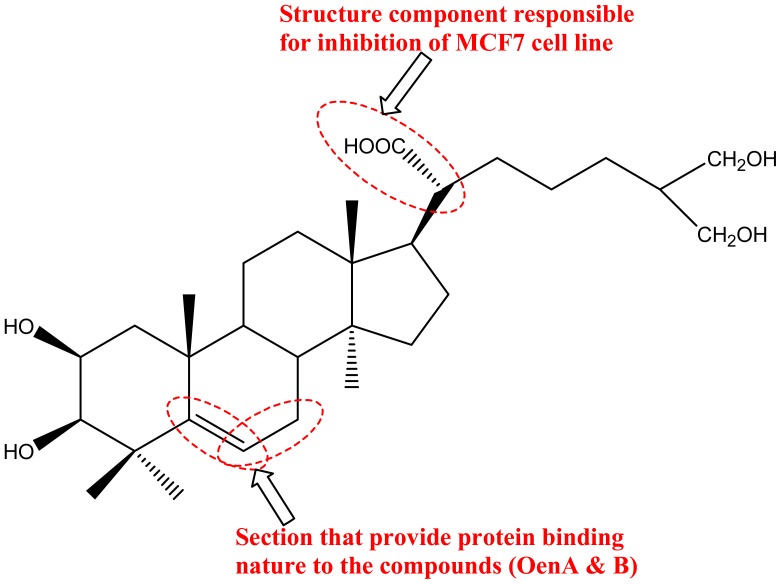
Representation of SAR for anti-MCF7 activity defined on the basis of inhibitors used in assay model building.

SAR analysis of experimentally validated molecules was performed with Dmax tool (http://dtai.cs.kuleuven.be/). It was based on hypothesis generated by 255 compounds used in assay model building. It was found that out of 255, 124 compounds act through its “non-hetero-non-aromatic” region and are responsible for the anti-MCF7 activity including OenA & B. Protein binding ability of studied compounds was identified by ToxTree software [10] (Figure 11).

### Possible target study for experimentally validated molecules

**Figure 12:**
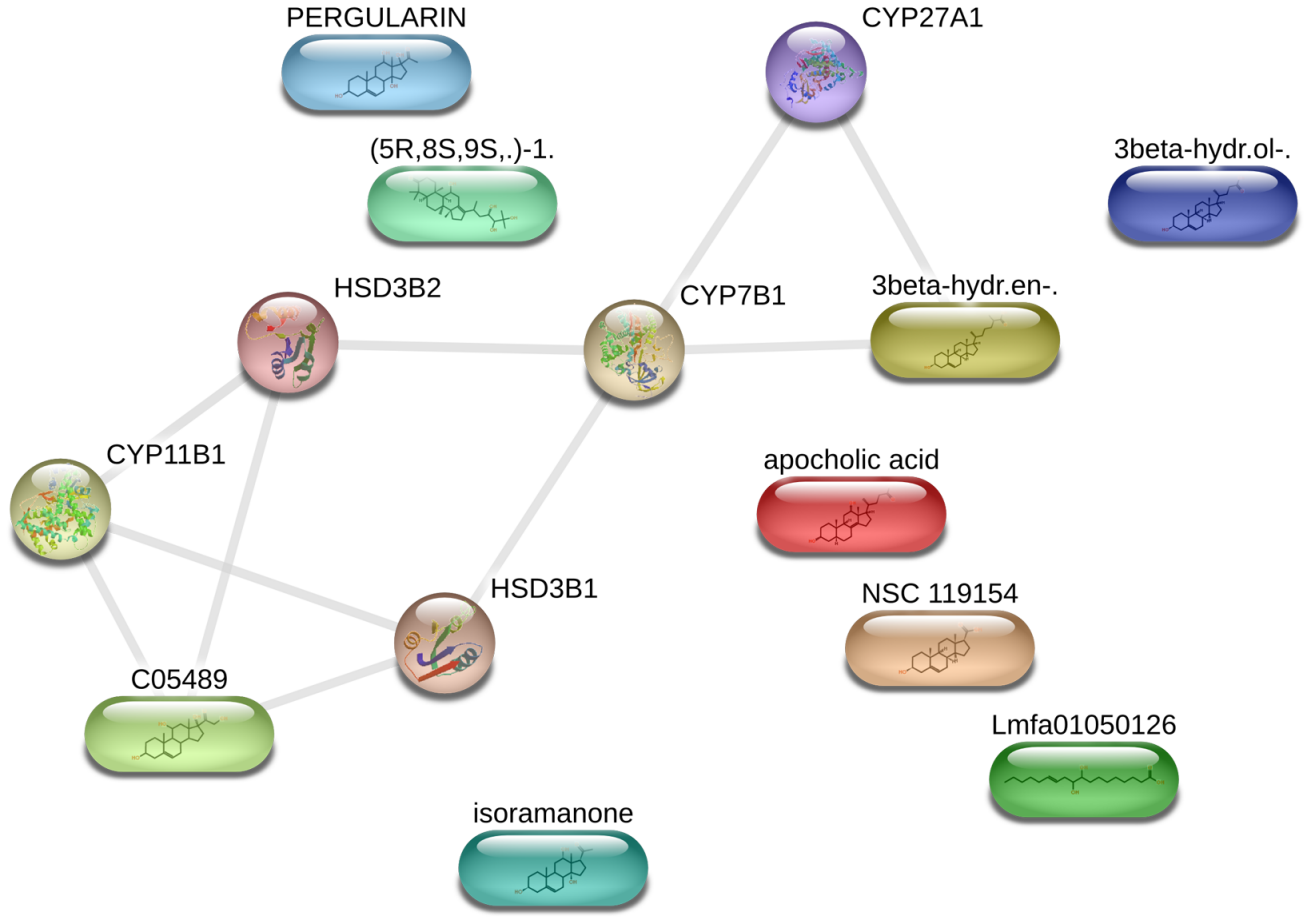
MetaDrug based putative target identification for exploring inhibitory activity of tetracyclic triterpenoid on MCF7 cancer cell line. System biology network (http://stitch.embl.de/) has been drawn for OenA & B based similar compounds.

MetaDrug database mining was performed to identify the possible existence of similar compounds, interaction and effects of compounds (OenA & B) in human model system. This database mining is based on experimental data available with the MetaDrug database. The data mining results have been categorized into following categories: (i) similarity search; (ii) possible metabolites of the query compounds generated by the different reactions in the body; (iii) probable pathway targets, where the query molecule may act; (iv) disease bio-markers concern with identified targets; (v) metabolic as well as toxicity networks where query molecule may act; and (vi) possible GO based localization, function and processes concern with query molecule. For screening process, similarity score threshold of 0.7 was selected. 28 compounds were matched with OenA, while 29 compounds were found matched with OenB. Best match compound was ‘3beta-Hydroxy-5-cholestenoate’ (with 89.29% and 90.36% structural similarity with OenA & B respectively). Since there was huge data collection based on pValue sorting of the data mining results, therefore interpretation with biological fact was used to analyze the predicted results of derived model. Predicted results showed compliance with prior studies, for example Zhang Y (2013) has reported that cytochrome P450 (CYP) plays a key role in metabolism and anticancer drug resistance of type-T triterpenoids [5]. Tetracyclic triterpenoid (e.g. androstenedione) also found in co-crystallized form with cytochrome P450 19A1 [11]. By data mining OenA & B showed structural similarity (80.68% of OenA and 81.61% of OenB) with CYP18 binding ligand ‘1-((8S,9S,10R,13S,14S,17S)-3-Hydroxy-10,13-dimethyl-2,3,4,7,8,9,10,11,12,13,14,15,16,17-tetradecahydro-1H-cyclopenta[a]phenanthren-17-yl)-ethanone’ with unspecified effect [12]. Compound OenB also showed structural similarity of 81.61% with ‘Dehydroepiandrosterone’ bound with CYP1A1 and ‘Pregnenolone’ bound with CYP2C19 [13], [14]. These results suggest that OenA and B have interaction ability with anticancer target enzyme (Aromatase: cytochrome P450 19A1). It was also found that the propene (C = CC) section of OenA & B provide the protein binding ability to the compounds (Figure 11). It is well known that the sub-cellular location of CYP is membrane/peripheral membrane protein (UniProt: P11511 (CP19A_HUMAN)) of the cell. Database mining results also suggested the gene ontology based locations of query molecules in the cell as, rough endoplasmic reticulum (pValue: 2.878e^−04^), plasma membrane (pValue: 7.741e^−04^), membrane (pValue: 8.183e^−04^), mitochondrial part (pValue: 1.140e^−02^), cell surface (pValue: 2.481e^−02^), plasma membrane (pValue: 4.294e^−02^), membrane (pValue: 5.400e^−02^), rough endoplasmic reticulum (pValue: 8.369e^−02^). Metabolic network study also suggests participation of OenA & B in steroid and related cellular metabolism (Figure 12). We concluded that, both OenA & B bind with CYP enzyme, which may result in increase in LDH level due to higher rate of oxidation of NADH for conversion of pyruvate to lactate. Ethanol-Inducible cytochrome P450 2E1 (4-Nitrophenol 2-Hydroxylase) regulates the response to oxidative stress and migration of breast cancer cells [15]. Anti-proliferative activity of compounds against MCF7 cell line is due to inhibition of aromatase targeting CYP19A1 [16].

### Docking study on Aromatase CYP19A1

**Figure 13:**
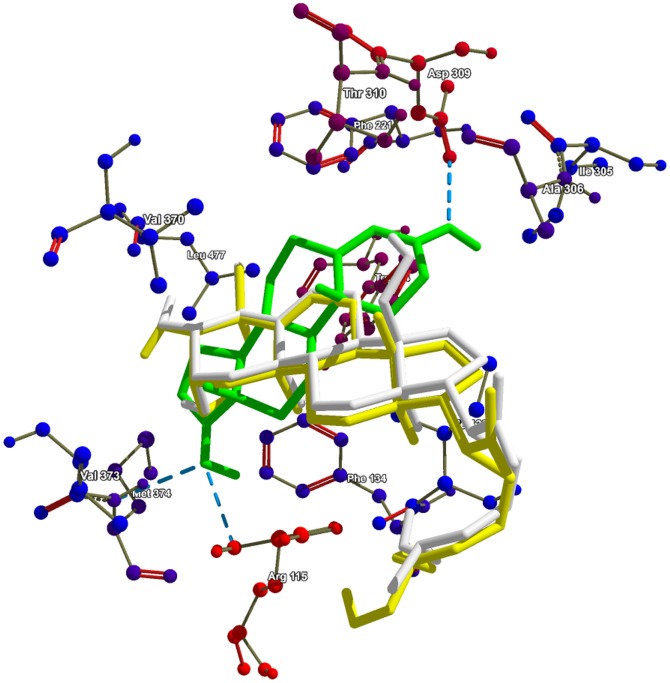
Conformations of query molecules (OenA & B) and androstenedione (control). Structurally identical OenA & B compounds has been shown with significantly similar docking pose. Note: Green color: androstenedione; Yellow color: OenA, White color: OenB.

**Figure 14:**
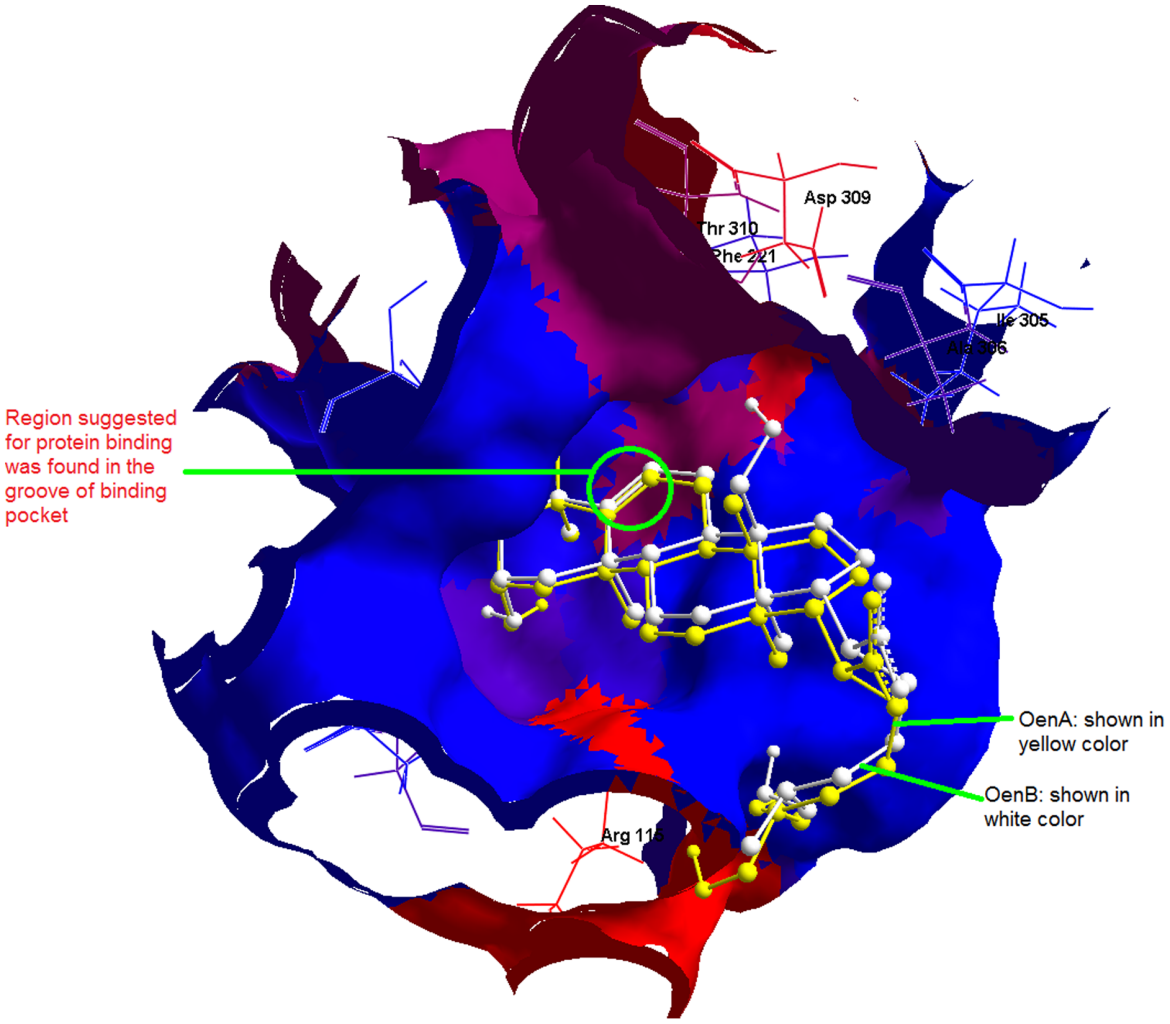
Docked view showed that the propene (C = CC) section of OenA & B provide the protein binding ability to the compounds was found inside the groove of binding pocket.

Out of the identified targets, human placental aromatase cytochrome P450 19A1 (PDB: 3EQM) was used for molecular docking studies. This protein's crystal structure contains androstenedione (a tetracyclic triterpenoid) in co-crystallized (PDB: 3EQM) form; therefore it was used as positive control in molecular docking study. The best docked control compound was found to be superimposed with co-crystallized ligand with binding affinity of −9.5 kcal/mol. Docking results showed nine conformations for each query molecule OenA & B. In case of OenA, eight out of nine conformations were found to be present at same location as of control compound (androstenedione), while in case of OenB, all conformations were clustered at the position similar to control compound. In comparison of control compound androstenedione, both OenA & B were found to be less interactive with CYP19A1 (about 41.05 to 46.31% less binding affinity). Common residues participating in molecular interaction were ARG-115, PHE-134, ILE-133, VAL-373, MET-374, VAL-370, TRP-224, THR-310, ALA-306, ILE-305, ASP-309, PHE-221, and LEU-477 (Table 5). It was observed that the control compound and OenB were interacting with CYP19A1 binding site through methionine (non-polar amino acid) & arginine (polar amino acid) & therefore forms hydrogen bonds with these residues. Similarly OenA was interacting with CYP19A1 binding site through non-polar amino acids leucine and isoleucine & therefore form hydrogen bond with these residues (Table 5) (Figure 13). In Figure 14, docked view showed that the propene (C = CC) section of OenA & B provide the protein binding ability to the compounds and it was found inside the groove of binding pocket.

**Table 5:** Molecular docking results of OenA and OenB.

Ligand	Binding Affinity (kcal/mol)	Interacting residues with H-bond length	Common binding site residues (amino acid & its position)
Androstenedione (control)	−9.5	MET-374 (2.97 Å), ARG-115 (3.18 Å)	ARG-115, ILE-133, PHE-134, PHE-221, TRP-224, ILE-305, ALA-306, ASP-309, THR-310, VAL-370, VAL-373, MET-374, LEU-477
OenA	−5.1	LEU-372 (3.08 Å), LEU-477 (3.01 Å), ILE-133 (2.91, 3.06 Å)	
OenB	−5.6	MET-374 (2.82 Å), ARG-115 (2.91 Å)	

## Discussion

Due to a complex parametric combination, development of computational assay model is a challenge. Model development means to mimic the *in vitro* bioassay into mathematical function. If functionality of the *in vitro* bioassay is known then we can draw a system diagram as ‘inputs followed by mathematical function and output. In *in silico* assay model function, inputs were, non-parametric independent components representing the structural information, concentration gradient as used in *in vitro* experiment and incubation time in hour. The *in silico* assay model function output was percentage inhibition. The *in silico* assay was a function of concentration gradient, treatment hour and independent components, which calculate the percentage inhibition in combination of these three components. The nonlinear relationship among ‘independent components’, concentration and time was established based on radial basis function. The radial basis function has property to be more resistant for noisy data; therefore RBF showed best efficiency for regression modeling over linear, polynomial and sigmoid kernel functions. The protocol has been implemented with LibSVM [6].

To develop an efficient non-linear model, first & most important step is sample selection. For designing of assay, it became necessary to sample the compounds with a wide spectrum of percentage inhibition. Initially, we collected complete redundant percentage inhibition assay data of 1318 compounds from CHEMBL for anti-proliferative activity against human cancer cell line MCF7. Since, we targeted to design an *in silico* assay for compounds belonging to tetracyclic triterpenoid family, therefore it was necessary to select those compounds which have fragmental similarity with tetracyclic triterpenoids. To put such structural constraint in the assay, we prepared a profile on the basis of molecular fingerprints of known tetracyclic triterpenoids. The profile was used to filter the initial raw compound set, for identification of MCF7 inhibitors, containing fragmental similarity with tetracyclic triterpenoids. This screening process was followed by removal of data redundancy. Finally 255 compounds from raw data were screened and selected for further *in silico* assay development. Thus, the developed assay model has an important feature, i.e. model first checked the “query compound” for its fragmental similarity with tetracyclic triterpenoids, before passing through model. For clearing this filter, the query molecules should lie within the similarity range (0.159154654 to 0.616295695) decided by the profile of reference tetracyclic triterpenoids. This step of fingerprint based compound selection provides a good applicability domain along with maintaining the diversity of compounds in training set. As shown in Figure 2, that the activity (percentage inhibition) of compound is varying from 0% to 100% in histogram. Each bar of the graph shows at least 10 compounds, which satisfy the statistical basis of sampling for model development.

In the model development and validation step, 255 MCF7 cell line growth inhibitors were used in *in silico* assay model development. The final developed model used 200 support vectors for decision making. Molecule's structural information was collected in the form of two-dimensional molecular descriptors. Since the independent factors are essential for model building, therefore principal components were derived from the set of descriptors. It was found that, except concentration and time, only 06 principal components were responsible factor for inhibition of MCF7 cell line. Since there was high dimensionality in the dataset, therefore it is necessary to normalize it. The independent components were normalized within the range of −1.0 to 1.0. The assay model function was established between 08 inputs and 1 output using LibSVM program [6]. The model function was optimized with two basic parameters ‘Gamma’ and ‘Cost’ along with RMSE calculation for each combination. Before prediction, it is essential to confirm the query molecules with the pharmacophore pattern of training set. This judgment has been made on the basis of molecular fingerprint based similarity and applicability domain check.

*In vitro* experiments were performed for anti-proliferative activity and LDH level testing with human cancer cell line MCF7 (wild type p53). P53 is seventh hall mark of cancer. It is a tumor suppressor [17]. By data mining results, it was hypothesized that query molecules showed their inhibitory activity through CYP leading to anti-proliferation. *In vitro* results showed that query molecules (OenA & B) have anti-proliferative activity as well as increased the level of LDH. Increased level of LDH indicates that mechanism of action of OenA & B is passing through Glycolysis pathway. In case of p53-induced apoptosis, the level of LDH get elevated along with other enzymes (Glyceraldehyde-3-phosphate dehydrogenase, enolase and pyruvate kinase) of anaerobic Glycolysis. To analyze the existence of anticancer activity, normal cell line (HaCaT) was used as control. The OenA & B did not show any anti-proliferative activity with HaCaT cell line. A comparison was made between *in silico* assay and *in vitro* bioassay results. Comparative study indicates that *in silico* assay results showed compliance with experimental results (Figure 9, 10). By compiling all results and reviews of literatures, it has been concluded that during the hypoxia condition (by incubation in CO_2_), cancer cell line don't tend towards apoptosis. But in presence of OenA & B, the cells tend towards normal cell growth from neoplastic condition; this shows the anti-proliferative nature of the OenA & B against cancer cell line MCF7. SAR analysis was performed on the basis of training set molecules. About 47% compounds of the training set showed similarity (fragment matching) with OenA & B. It was found that ‘carboxylic acid group’ along with pentacyclic non-aromatic ring is being the reason for inhibitory activity of OenA & B against MCF7 cell line. Besides it, SN2.SN2-nucleophilic aliphatic substitution was also found important parameter to provide the protein binding potential into the compounds (OenA & B). The modeling work has been performed with human breast cancer cell line MCF7. MCF7 cell line has wild type p53. Under the normal oxygen level condition, p53 induced apoptosis proceed through caspases by mitochondrial cytochrome C. It is reported that MCF7 cells have wild type p53 function along with property of tumor cell generation, but do not proceed for p53 dependent apoptosis [18]. In another report, growth inhibition of breast cancer cell by progesterone which is structurally similar to OenA & B has been described. It has been stated that the inhibition is due to induction of cell differentiation, but not apoptosis. Progesterone significantly enhances the apoptosis in MCF7, over expressing p53, but not in control cells [19]. In hypoxia condition, HIF-1α gene get activated to support the cells for anaerobic respiration using lactate dehydrogenase-A. Crystallographic structure of a tetracyclic triterpenoid named androstenedione is recently reported, bound with cytochrome P450 (PDB: 3EQM), which also support the hypotheses. It is reported that aromatase cytochrome P450 is an endoplasmic reticulum enzyme and perform three reactions for C19-methy hydroxylation and aromatization of steroid ring. MetaDrug database (Thomson Reuter, 2014, USA) mining results also suggest the metabolite generation for OenA & B by similar reaction of aliphatic hydroxylation. Therefore, we concluded that OenA & B initially interact with CYP to enhance the conversion of NADPH to NADP^+^. We hypothesized that OenA & B bind well to CYP in MCF7 cell line, and this step helps in down regulation of endogenous p53 protein, ultimately resulting into growth inhibition of MCF7 cells due to induction of cell differentiation. In support of our hypothesis, similar mechanism of action also reported by Alkhalaf and El-Mowafy, 2003 [19].

## Supporting Information

Appendix S1 **Contains structure elucidation data for compounds used in experimental validation.**
(DOCX)

Data S1 **Raw collected data from CHEMBL.**
(XLS)

Data S2 **Tetracyclic triterpenoids collected from ChEBI database.**
(TSV)

Data S3 **Non-redundant compounds used in model development.**
(XLSX)

Data S4 **Comparative data for kernel function evaluation with given dataset.**
(XLSX)

Data S5 **Cost and gamma parameter combinations for model optimization.**
(XLSX)

Data S6 **Details about 200 support vectors using in model.**
(TXT)
