# Supplementary material for: In Silico Assay Development for Screening of Tetracyclic Triterpenoids as Anticancer Agents against Human Breast Cancer Cell Line MCF7
Source: PLoS One. 2014 Nov 3;9(11):e111049. doi: 10.1371/journal.pone.0111049 (PMC4218838; doi:10.1371/journal.pone.0111049)
Supplement: Appendix S1 — Contains structure elucidation data for compounds used in experimental validation. (DOCX) [file pone.0111049.s001.docx]

**Appendix S1**

Compounds used in experimental validation

*Oenotheranstrol-A.* Lanosta-5-en-2β, 3β,26,27-tetraol-21-oic acid (Oenotheralanosterol A, 1):White solid, m.p. 210—212 ℃; R_f_ 0.51 (MeOH∶H_2_O,1∶1), [α]_D_^22^ －32.20 (c 0.5, MeOH); UV (MeOH) λmax: 236, 271, 346; IR (KBr) νmax:3510, 3465, 3436, 3240. 2929, 2880, 1702, 1633, 1455, 1382, 1272, 1193, 1173, 1145, 1034, 934 cm^－1^; EI MS m/z (rel. int.): 506 [M]^＋^(C_30_H_50_O_6_) (1.5), 331 (16.3),313 (17.2), 301 (5.1), 295 (12.8), 286 (8.9), 280 (9.1), 236 (10.2), 215 (10.5), 208 (10.4), 193 (3.5), 163 (4.9),135 (14.3), 107 (9.8), 95 (11.8), 90 (100), 86 (9.5), 83(21.2); ESI Mass (positive mode) (M＋Na)^＋^: m/z 529; Anal. calcd for C_30_H_50_O_6_: C 71.14, H 9.88, O 18.97;found C 69.90, H 10.50; ^1^H NMR (CD_3_OD, 300 MHz): δ 1.58 (d, J = 13.6 Hz, 1H, H-1), 2.68 (d, J = 5.3 Hz, 1H, H-1), 3.83 - 3.87 (m, 1H, H-2), 3.52 (d, J = 10.8 Hz, 1H, H-3), 5.26 (br s, 1H, H-6), 2.30 (dd, J = 10.3, 5.6 Hz, 1H, H-7), 1.90 (dd, J = 5.6, 5.3 Hz, 1H, H-7), 1.65 - 1.69 (m, 1H, H-8), 1.80 -1.83 (m, 1H, H-9), 1.76 (ddd, J = 13.3, 8.2, 8.1 Hz, 1H, H-11), 1.70 (ddd, J = 8.2, 8.1, 5.3 Hz, 1H, H-11), 2.65 (ddd, J = 13.3, 8.1, 6.5 Hz, 1H, H-12), 1.76 (ddd, J = 8.1, 6.5, 5.2 Hz, 1H, H-12), 1.87 (ddd, J = 12.9, 8.1, 6.2 Hz, 1H, H-15), 2.01 (ddd, J = 8.1, 6.2, 5.2 Hz, 1H, H-15), 1.53 - 1.98 (m, 2H, H-16), 2.66 - 2.70 (m, 1H, H-17), 1.14 (br s, 3H, H-18), 1.34 (s, 3H, H-19), 2.71 - 2.74 (m, W1/2 6.5 Hz, 1H, H-20), 1.98 - 2.28 (m, 2H, H-22), 1.51-1.55 (m, 2H, H-23), 1.49 - 1.68 (m, 2H, H-24), 2.65 - 2.69 (m, 1H, H-25), 3.34 (d, J = 12.0 Hz, 1H, H-26), 3.30 (d, J = 12.0 Hz, 1H, H-26), 3.61 (d, J = 11.3 Hz, 1H, H-27), 3.55 (d, J = 11.3 Hz, 1H, H-27), 0.78 (br s, 3H, H-28), 1.01 (br s, 3H, H-29), 1.19 (br s, 3H, H-30); ^13^C NMR (CD_3_OD, 75 MHz) : δ 33.80 (C-1), 67.38 (C-2), 78.77 (C-3), 41.14 (C-4), 139.81 (C-5), 128.60 (C-6), 29.62 (C-7), 44.35 (C-8), 48.51 (C-9), 39.29 (C-10), 33.84 (C-11), 27.35 (C-12), 42.88 (C-13), 42.64 (C-14), 32.97 (C-15), 24.84 (C-16), 50.01 (C-17), 17.74 (C-18), 24.79 (C-19), 43.40 (C-20), 182.48 (C-21), 42.27 (C-22), 19.24 (C-23), 25.33 (C-24), 47.97 (C-25), 68.47 (C-26), 71.41 (C-27), 16.38 (C-28), 29.25 (C-29), 17.41 (C-30).

*Oenotheranstrol-B.* Lanosta-5-en-2β,3β,26,30-tetrol-21-oic acid (Oenotheralanosterol B, 2): White solid, m.p. 212—214 ℃; Rf 0.48 (MeOH∶H_2_O,1∶1); [α]_D_^22^ －38.4 (c 0.4, MeOH); UV (MeOH) λmax:232, 267, 351;; IR (KBr) νmax:3515, 3480, 3442, 3250, 2927, 2855, 1694, 1633, 1454, 1391, 1273, 1150, 1110 cm^－1^; EI MS m/z (rel. int.): 506[M]＋(C30H50O6) (1.1), 390 (10.3), 347 (15.1), 338 (6.9),332 (7.3), 324 (14.8), 314 (18.2), 302 (11.5), 296 (11.4),266 (24.3), 236 (6.1), 206 (5.2), 193 (4.1), 168 (4.2),164 (4.3), 159 (4.9), 135 (7.8), 98 (20.2), 91 (100), 83(14.7), 76 (23.2); ESI Mass (positive mode) [M＋Na]^＋^: m/z 529; Anal. Calcd. for C_30_H_50_O_6_: C 71.14, H 9.88, O18.97; found C 70.69, H 10.69; ^1^H NMR (CD_3_OD, 300 MHz ) δ: 1.51 (d, J = 13.2 Hz, 1H, H-1), 2.68 (d, J = 5.2 Hz, 1H, H-1), 3.70 - 3.72 (m, 1H, H-2), 3.36 (d, J = 9.5 Hz, 1H, H-3), 5.23 (br s, 1H, H-6), 1.96 - 2.22 (m, 2H, H-7), 1.64 -1.68 (m, 1H, H-8), 1.80 - 1.84 (m, 1H, H-9), 1.68 - 1.79 (m, 2H, H-11), 1.80 - 2.66 (m, 2H, H-12), 1.93 – 2.07 (m, 2H, H-15), 1.51 - 1.96 (m, 2H, H-16), 2.16 - 2.20 (m, 1H, H-17), 1.04 (br s, 3H, H-18), 1.34 (s, 3H, H-19), 2.84 - 2.87 (m, 1H, H-20), 1.32 – 1.62 (m, 2H, H-22), 1.35-1.67 (m, 2H, H-23), 1.29-1.65 (m, 2H, H-24), 1.90 – 1.94 (m, 1H, H-25), 3.52 (d, J = 10.8 Hz, 1H, H-26), 3.48 (d, J = 10.8 Hz, 1H, H-26), 0.89 (d, J = 5.7 Hz, 3H, H-27), 0.69 (br s, 3H, H-28), 0.96 (br s, 3H, H-29), 3.24 (br s, 1H, H-30), 3.30 (br s 1H, H-30); ^13^C NMR (CD_3_OD, 75 MHz) : δ33.77 (C-1), 69.82 (C-2), 78.27 (C-3), 40.92 (C-4), 142.29 (C-5), 123.52 (C-6), 29.30 (C-7), 48.27 (C-8), 50.01 (C-9), 39.12 (C-10), 25.46 (C-11), 29.17 (C-12), 44.27 (C-13), 43.57 (C-14), 38.27 (C-15), 31.93 (C-16), 54.50 (C-17), 18.01 (C-18), 24.30 (C-19), 40.56 (C-20), 181.09 (C-21), 48.17 (C-22), 19.21 (C-23), 24.61 (C-24), 48.99 (C-25), 68.40 (26), 14.10 (C-27), 17.84 (C-28), 21.76 (C-29), 66.37 (C-30).
